# Spectrum of hepatitis B and hepatitis C-related cancers in India

**DOI:** 10.3332/ecancer.2024.1760

**Published:** 2024-09-06

**Authors:** Sivaranjini Kannusamy, Amey Oak, Sandhya Cheulkar, Kamesh Maske, Esha Dashmukhe, Ashwini Patil, Manisha Morajkar, Manju Sengar, Ganesh Balasubramaniam, Rajesh Dikshit

**Affiliations:** 1 Homi Bhabha National Institute, Mumbai 400094, India; 2 Division of Cancer Care, Hospital Cancer Registries and Survival Studies, Centre for Cancer Epidemiology, Tata Memorial Centre, Mumbai 410210, India; 3 Department of Medical Oncology, Tata Memorial Centre, Mumbai 410210, India; a https://orcid.org/0000-0001-7414-8751; b https://orcid.org/0009-0004-1893-4191

**Keywords:** cancer, hepatitis B, hepatitis C

## Abstract

**Introduction:**

Hepatitis-B virus infection contributes to 40%–50% of the Hepato-cellular carcinomas (HCC) in India, while hepatitis-C virus infection accounts for 12%–32% of cases. This study aimed at determining the patterns of cancers among patients with hepatitis B and C.

**Materials and methods:**

This was a retrospective study of cancer patients with histologically proven diagnoses of cancer registered at Tata Memorial Hospital in Mumbai between 2017 and 2018. The proportional incidence ratio (PIR) was computed by dividing the observed number of site-specific cancer cases by the expected number.

**Results:**

The study participants’ mean (SD) age was 48.69 (±16.91) years with a male-to-female ratio of 1.36. The prevalence of hepatitis B and C was 1.93% and 1.17%, respectively. Liver cancer showed the highest occurrence rate with notably increased PIR among individuals positive for hepatitis B (males: 14.41, females: 10.89) and hepatitis C (males: 7.15, females: 10.42). Furthermore, hepatitis B-positive patients showed elevated PIR for haemato-lymphoid malignancies such as multiple myeloma and non-Hodgkin’s lymphoma.

**Limitation:**

The correlation between HBsAg and specific cancer types (PIRs) is limited by small case numbers, requiring careful interpretation of these findings.

**Implications and conclusion:**

The PIR for liver cancer was heightened in both hepatitis B and C patients. Strengthened surveillance, including pre-screening for hepatitis B and C positive infection among cancer patients, as well as screening for HCCs among hepatitis seropositive individuals, is crucial to mitigate the incidence of HCC.

## Introduction

Cancer is responsible for one out of every six fatalities worldwide, or about 10 million deaths. Cancer-causing infections such as human papillomavirus and hepatitis cause one-third of all cancer cases in low and lower-middle-income nations [1].

Infection with the hepatitis B Virus (HBV) predominantly damages the liver, posing a significant risk of mortality from cirrhosis and hepatocellular cancer. Around 296 million individuals worldwide have chronic hepatitis B, and 820,000 died in 2019 as a result of cirrhosis and hepatocellular cancer [2]. In India, the prevalence of HBV infection ranges between 3.2% and 4.2% [3]. 

The most frequent histologic type is hepatocellular carcinoma (HCC), which accounts for 80% of all primary liver cancers [4]. In India, 40%–50% of HCC are caused by HBV infection, whereas 12%–32% are caused by hepatitis C Virus (HCV) infection [3]. HBV and HCV viruses have been found in a variety of extra-hepatic tissues, suggesting that they may speed up the oncogenesis of extra-hepatic cancers. Chronic HBV infection has been linked to a variety of extrahepatic malignancies, including gastric cancer, pancreatic cancer, intra- and extra-hepatic bile duct carcinoma, non-Hodgkin lymphoma (NHL), breast cancer, thyroid cancer, lung cancer and skin cancer [5]. HCV has been identified in extrahepatic organs and may, therefore, directly induce carcinogenesis by prolonged inflammation and oncogenesis of target tissues. Cholangiocarcinoma, pancreatic adenocarcinoma, papillary thyroid cancer, oral squamous cell cancer and renal cancer are the five most often reported non-HCC cancers associated with HCV [6, 7]. 

The lack of comprehensive data on the spectrum of malignancies among HBV and HCV patients in India underscores a critical gap in addressing these infections and developing effective prevention and management strategies. Tata Memorial Hospital (TMH), India’s largest tertiary referral cancer centre, plays a crucial role in addressing this issue. TMH, which treats patients from across the country, provides unique insights into cancer patterns among these groups. This study conducted at TMH focused on analyzing cancer patterns among HBV and HCV patients in Mumbai, India, from 2017 to 2018. Understanding the prevalent types of cancers associated with these infections can guide policies related to vaccination, screening, early detection and antiviral therapies. TMH’s findings not only benefit India but also offer valuable references globally for managing and preventing cancers linked to HBV and HCV. 

## Methodology

The current study was a retrospective study of cancer patients with histologically proven diagnosis of cancer registered at TMH in Mumbai from 2017 to 2018. After pre-test counseling, all cancer patients, both existing and new, presenting to TMH for diagnosis/treatment underwent screening for viral markers including hepatitis B surface antigen (HBsAg) and HCV. We specifically calculated the proportional incidence ratio (PIR), for cancer patients who tested positive for HBsAg using ELISA. Those individuals who tested positive for IgM anti-HBc antibodies and anti-HBs antibodies were categorized as HBsAg negative. This classification was grounded in the recognition that acute hepatitis B can manifest without transitioning into chronic infection in the majority of cases.

Cancer patients found positive for HBsAg or HCV testing at TMH are referred to the hospital’s gastrointestinal clinic. Each cancer patient has a distinct, comprehensive case file containing information about their sociodemographic characteristics, laboratory results and treatment regimens documented by the treating physician in the electronic medical record (EMR). Age, sex, residence, education, anatomic location, stage, histology of cancer and specifics of viral markers including HBsAg, HCV and HIV infection are abstracted from the EMR and uploaded into the hospital’s internal software. The International Classification of Diseases for Oncology [8] is employed by coding experts to classify cancer sites accurately.

The analysis of the data was performed in Microsoft Excel. We estimated the anticipated number of site-specific cancers among HBsAg and HCV-positive cancer cases utilizing the gender and age-specific proportions of each cancer site which were recorded in the Hospital Cancer Registry of TMH, Mumbai for the years 2017 and 2018. By dividing the overall number of cancer cases in each age group within the study data set by the corresponding age and site-specific proportions in the standard, the expected number of instances of a given cancer was calculated. To calculate the PIR, the observed number of site-specific cancer cases among HBsAg and HCV-positive cancer cases was divided by the expected number [9]. Since this study relied on hospital cancer data, only age-specific rates were calculated. Age-standardized rates necessitate population rates for calculation. A higher PIR indicates that, in HBsAg and HCV-positive cancer cases, the proportion of cancer at a given cancer site is higher than would be predicted based on the information from TMH’s hospital-based cancer registry. Under the presumption of a Poisson distribution, the estimated standard error of the PIR was used to calculate 95% confidence ranges for the observed instances. Each two-sided PIR test was deemed significant at a 5% level, and all tests were two-sided.

## Results

A total of 71,001 cancer patients were registered during the period 2017 to 2018 in TMH, Mumbai. Table 1 describes the demographic characteristics of the patients and viral markers. The mean (SD) age of the study participants was 48.69 (±16.91) with nearly one-third being elderly. The male-to-female ratio was 1.36 in our study. Approximately (38.79%) of cancer patients hailed from the western region of India. The proportion of participants seropositive for HBsAg, HCV and both were 1,367 (1.93%), 831 (1.17%) and 34 (0.05%), respectively. The proportion of HIV-positive cancer patients was 418 (0.59%). A total of 523 patients who tested positive for HBsAg received antiviral treatment after consulting the hepatology clinic at TMH, while the treatment status of the remaining patients is unknown.

Table 2 outlines the cancer sites among HBsAg-positive cancer cases within the study group and provides estimates of PIR for the various cancer sites in males and females. Liver cancer emerged as the commonest site among HBsAg-positive cancer cases in both genders. The PIR for liver cancer was notably high in males at 14.41 (95% CI, 13.0–15.9), and in females at 10.89 (95% CI, 7.65–15.4), overall PIR of the liver was 16.31 (95% CI, 14.7–17.9). In males, there were elevated PIRs greater than 1 for nasopharynx, small intestine, mesothelioma, breast, renal pelvis, eye, secondary respiratory and digestive organs, as well as NHL and multiple myeloma. However, these increases were not statistically significant. For females, elevated PIRs greater than 1 were observed for the lip, nasopharynx, esophagus, colon, rectum, anus, other thoracic organs, bone, skin melanoma, soft tissue and peripheral nervous system, vulva, cervix, corpus uteri, ovary, other female genital organs, renal pelvis, other skin, as well as secondary respiratory and digestive organs. However, similar to males, these increases were not statistically significant.

Table 3 illustrates the cancer sites among HCV-positive cancer cases in the study population and shows the estimates of PIR for the various cancer sites in males and females. The PIR was significantly higher for liver cancer in males 7.15 (95% CI, 5.79–8.84) and in females, 10.42 (95% CI, 7.28–14.90) and overall PIR was 7.88 (95% CI, 6.57–9.45). Elevated PIRs greater than 1 were observed in various cancer sites, including lip, other oropharynx, esophagus, stomach, small intestine, colon, rectum, anus, gall bladder, melanoma, mesothelioma, retroperitoneum, vulva, corpus uteri, ovary, urinary bladder, other endocrine glands, secondary lymph nodes, secondary respiratory and digestive organs, other secondary organs, Hodgkin’s disease, NHL and other unspecified sites. However, these increases were not found to be statistically significant.

## Discussion

This study details the prevalence of different cancer sites among individuals who are positive for HBsAg and HCV. We evaluated PIR for various cancer sites for HBsAg and HCV-positive cases seeking care at TMH using age and gender-specific proportions of each cancer site from the Hospital Cancer Registry of TMH as a reference. The prevalence of HBsAg and HCV positivity in our study was 1.93% and 1.17%, respectively.

Infection with HBV is widely known as a risk factor for HCC [10]. Our findings revealed that the PIR was considerably higher for HCC in persons who were seropositive for HBsAg and HCV when compared to the data from TMH’s hospital cancer registry. In both genders, HBsAg seropositive subjects also had higher PIR for certain non-liver cancers such as multiple myeloma, NHL, leukemia and Hodgkin lymphoma. Our findings on the association between HBsAg with HCC, leukemia and lymphoma align with a large case-control study conducted among the Chinese population. A case-control study observed significant associations between HBsAg and HCC, gastrointestinal cancers, NHL and leukemia [10]. A population-based cohort study in China and a few other prospective studies also established an association of HBsAg with liver cancer and non-liver cancer especially digestive system cancers, oral cancers and lymphoma [11–13]. Studies across the globe have shown strong evidence of an association between HCV and HCC [14]. Our study findings were also consistent with elevated PIR for HCC among HCV seropositive patients.

Our study findings suggest a significant association between HCC and hepatitis B and C infection. However, the elevated PIR for hematological malignancies among HBsAg-positive individuals may stem from an additional mandatory investigation of HBsAg core and envelope antigens in patients with hematological cancer. Clinicians managing hepatitis B and C infection-positive patients should remain vigilant for malignancies. The National Viral Hepatitis Control Program recommends routine HCC surveillance with abdominal ultrasound and alpha-fetoprotein testing every 6 months for individuals with cirrhosis, a family history of HCC, an age of more than 40 years and an elevated HBV DNA level of more than 2,000 IU/mL [15]. Further reactivation of the HCV in patients receiving chemotherapy has resulted in a deleterious clinical course. So, universal pre-chemotherapy HCV testing for patients with hematological malignancies is recommended by current guidelines [16]. Strengthening surveillance by pre-screening for hepatitis B and C positive infection among cancer patients as well as HCCs among hepatitis seropositivity is important to reduce the incidence of HCC. The paper also highlights the crucial role of addressing HBV and HCV treatments in managing cancer risks, particularly due to their well-established links with specific cancers such as HCC and liver cancer. It stresses the importance of managing viral infections to decrease associated cancer risks, emphasizing the significance of screening, early detection and prompt treatment to prevent the development of these cancers.

Among all cancer sites, the prevalence of HIV infection was 0.5%. NHL was the most common cancer among HIV-positive patients accounting for 108 (26.09%). In female HIV-positive cancer patients, cervical cancer was the leading type, representing 70 (42.17%). Among males, mouth cancer was the most prevalent, comprising 42 (16.94%). These findings underscore the importance of targeted cancer screening and prevention strategies for HIV-positive individuals.

Our study demonstrates several significant strengths. First, we analyzed a substantial dataset of 710,001 cancer cases, enabling a comprehensive exploration of the relationship between HBsAg and HCC. This extensive dataset also facilitated an examination of how HBsAg and HCV relate to various cancer types, offering a broad perspective on viral influences in cancer development. Additionally, our adherence to standardized guidelines for evaluating seromarker prevalence ensures the reliability and consistency of our findings. A key contribution of our research is confirming HBsAg and HCV infections as notable risk factors for liver cancer (HCC). By establishing this connection, we emphasize the importance of viral screening and effective management strategies to reduce the burden of liver cancer, particularly in regions where these infections are prevalent.

However, despite these strengths, our study faces several limitations that merit consideration. The retrospective nature of data collection, relying on hospital records to ascertain HBsAg and HCV status, introduces potential biases and challenges in establishing temporal relationships between viral infections and carcinogenesis. It is difficult to determine definitively whether HBsAg or HCV infection preceded the development of cancer in these cases. Retrieving hepatitis treatment information from the EMR is challenging for patients receiving care from facilities outside of TMH. Moreover, because our study was conducted within a hospital setting, we faced limitations in calculating standardized rates, which are more robust for comparisons across different populations. Furthermore, some of our statistical analyses were constrained by small case numbers in certain cancer sites (PIRs), necessitating cautious interpretation of these findings.

## Conclusion

In our study, the prevalence of hepatitis B and C was 1.9% and 1.10%, in all cancer sites, respectively. The PIR was higher for liver cancer across both genders in cancer patients who tested positive for HBsAg and HCV. The necessity for primary and secondary preventive efforts to decrease the incidence of HCC is highlighted by the elevated PIR for liver cancer among HBsAg and HCV patients.

## Conflicts of interest

There are no conflict of interest to declare.

## Funding

This study received no funding.

## Informed consent

The database of the hospital-based cancer registry is securely maintained by authorized study personnel. Measures have been ensured to safeguard the user ID and password for accessing the data ensuring the patient rights are upheld and protected.

## Author contributions

- Dr Sivaranjini Kannusamy: Conceptualization, design, statistical analysis, data interpretation and manuscript writing.
- Dr Amey Oak: Provided knowledgeable direction and oversight to ensure the study’s methodological rigor and contributed to manuscript writing.
- Mrs Sandhya Cheulkar: Contributed to data validation and interpretation.
- Dr Kamesh Maske: Contributed to the conceptualization and manuscript writing.
- Mrs Esha Dashmukhe, Mrs Ashwini Patil and Ms Manisha Morajkar: Contributed to data abstraction and validation.
- Dr Manju Sengar: Made substantial contributions in drafting the final report.
- Dr Ganesh Balasubramaniam: Substantial contributions to the study administration and conceptualization.
- Dr Rajesh Dikshit: Contributed to Conceptualization, design, management of the study and assisted in drafting the final report.

## Figures and Tables

**Table 1.** Characteristics of cancer patients registered at TMH, Mumbai, India. Year: 2017–2018.

S.NO	Characteristics	Total *n* (%)
	Total cases	71,001
1.	**Age in years**	
	0–19	4,856 (6.84)
	20–39	12,672 (17.85)
	40–59	32,437 (45.69)
	60 and above	21,036 (29.63)
2.	**Gender**	
	Male	40,928 (57.64)
	Female	30,073 (42.36)
3.	**Education**	
	Illiterate	10,623 (15.19)
	Literate	1,554 (2.22)
	Primary & middle	24,644 (35.24)
	Secondary	18,209 (26.03)
	Graduates	14,605 (20.88)
	Unknown	293 (0.41)
4.	**Region**	
	North	1,057 (1.49)
	South	966 (1.36)
	East	21,596 (30.42)
	West	27,541 (38.79)
	Central	15,467 (21.78)
	North-east	2,634 (3.71)
	Foreign	1,740 (2.45)
5.	**HBsAg status**	
	Positive	1,367 (1.93)
	Negative	68,534 (98.07)
6.	HCV status	
	Positive	831 (1.17)
	Negative	70,223 (98.82)
7.	**HBsAg and HCV status**	
	Positive	34 (0.05)
	Negative	70,958 (99.95)
8.	**HIV status**	
	Positive	418 (0.59)
	Negative	70,587 (99.41)

**Table 2.** Details of cancer sites and PIR for the cancer sites in the 1,367 patients with HBsAg and cancer registered at TMH, Mumbai, India. Year: 2017–2018.

ICD-10	SITE	Male				Female				Total			
		Observed number	Expected number	PIR	95% CI	Observed number	Expected number	PIR	95% CI	Observed number	Expected number	PIR	95% CI
C00	Lip	1	5.59	0.18	0.03-1.27	1	0.73	1.37	0.19-9.74	2	5.53	0.36	0.09-1.44
C01-C02	Tongue	36	80.00	0.45	0.32-0.62	5	8.12	0.62	0.25-1.47	41	75.16	0.55	0.40-0.74
C03-C06	Mouth	69	145.06	0.48	0.38-0.60	5	12.75	0.39	0.16-0.94	74	132.89	0.56	0.44-0.69
C07-C08	Saliv Gland	1	5.08	0.20	0.03-1.39	1	1.11	0.90	0.12-6.39	2	5.80	0.35	0.08-1.37
C09	Tonsil	5	7.58	0.66	0.27-1.59	-	-	-	---	5	6.66	0.75	0.31-1.80
C10	Oth Oropharynx	3	7.60	0.39	0.13-1.22	-	-	-	---	3	6.64	0.45	0.14-1.40
C11	Nasopharynx	6	5.96	1.01	0.45-2.24	1	0.86	1.16	0.16-8.22	7	6.05	1.16	0.55-2.42
C12-C13	Hypopharynx	10	18.80	0.53	0.29-0.98	1	1.77	0.56	0.07-4.00	11	17.42	0.63	0.34-1.13
C14	Pharynx	-	-	-	---	-	-	-	---	-	-	-	---
C15	Oesophagus	17	32.04	0.53	0.33-0.85	9	7.68	1.17	0.61-2.25	26	37.70	0.69	0.46-1.01
C16	Stomach	33	38.58	0.86	0.61-1.20	7	7.61	0.92	0.43-1.92	40	42.59	0.94	0.68-1.28
C17	Small Intestine	4	3.83	1.05	0.39-2.79	-	-	-	---	4	3.99	1.00	0.37-2.67
C18	Colon	10	25.82	0.39	0.21-0.72	6	5.69	1.05	0.47-2.34	16	29.53	0.54	0.33-0.88
C19-C20	Rectum	28	33.82	0.83	0.57-1.20	10	7.14	1.40	0.75-2.60	38	38.14	1.00	0.72-1.36
C21	Anus	1	3.75	0.27	0.04-1.89	1	0.73	1.37	0.19-9.74	2	4.12	0.49	0.12-1.94
C22	Liver	364	25.27	14.41	13.0-15.9	31	2.85	10.89	7.65-15.4	395	24.22	16.31	14.7-17.9
C23-C24	Gall Bladder	38	37.27	1.02	0.74-1.40	23	23.64	0.97	0.64-1.46	61	69.14	0.88	0.68-1.13
C25	Pancreas	16	17.16	0.93	0.57-1.52	2	3.88	0.52	0.12-2.06	18	19.79	0.91	0.57-1.44
C26	Oth Dig Org	-	-	-	---	-	-	-	---	-	-	-	---
C30-C31	Nose & Sinus	4	6.67	0.60	0.23-1.60	-	-	-	---	4	7.09	0.56	0.21-1.50
C32	Larynx	25	27.23	0.92	0.62-1.35	-	-	-	---	25	22.20	1.13	0.76-1.66
C33-C34	Lung	66	84.53	0.78	0.61-0.99	12	14.70	0.82	0.46-1.43	78	89.93	0.87	0.69-1.08
C37-C38	Oth Thorac Org	2	2.54	0.79	0.19-3.14	1	0.19	5.25	0.73-37.2	3	2.27	1.32	0.42-4.09
C40-C41	Bone	12	19.88	0.60	0.34-1.06	7	4.27	1.64	0.78-3.43	19	22.55	0.84	0.53-1.32
C43	Melanoma of Skin	-	-	-	---	1	0.66	1.51	0.21-10.7	1	3.25	0.31	0.04-2.18
C44	Other Skin	5	8.86	0.56	0.23-1.35	4	2.49	1.61	0.60-4.28	9	11.05	0.81	0.42-1.56
C45	Mesothelioma	2	1.13	1.77	0.44-7.06	-	-	-	---	2	1.10	1.82	0.45-7.28
C47&C49	Soft tissue & PNS	13	25.92	0.50	0.29-0.86	11	6.66	1.65	0.91-2.98	24	31.27	0.77	0.51-1.14
C48	Retroperitoneum	2	2.54	0.79	0.19-3.14	-	-	-	---	2	3.23	0.62	0.15-2.47
C50	Breast	4	2.99	1.34	0.50-3.55	70	86.73	0.81	0.63-1.02	74	151.31	0.49	0.38-0.61
C51	Vulva	-	-	-	---	2	0.90	2.23	0.55-8.92	2	1.54	1.30	0.32-5.19
C52	Vagina	-	-	-	---	1	2.50	0.40	0.05-2.84	1	4.29	0.23	0.03-1.65
C53	Cervix	-	-	-	---	37	32.46	1.14	0.82-1.57	37	55.78	0.66	0.48-0.91
C54-C55	Corpus Uteri	-	-	-	---	10	9.39	1.06	0.57-1.97	10	16.13	0.62	0.33-1.15
C56	Ovary etc	-	-	-	---	27	20.51	1.32	0.90-1.91	27	35.23	0.77	0.52-1.11
C57	Oth Female Gen Org	-	-	-	---	1	0.18	5.58	0.78-39.5	1	0.31	3.25	0.45-23.0
C58	Placenta	-	-	-	---	-	-	-	---	-	-	-	---
C60	Penis	5	7.32	0.68	0.28-1.64	-	-	-	---	5	5.60	0.89	0.37-2.14
C61	Prostate	17	40.22	0.42	0.26-0.67	-	-	-	---	17	30.77	0.55	0.34-0.88
C62	Testis	8	11.80	0.68	0.33-1.35	-	-	-	---	8	9.03	0.89	0.44-1.77
C63	Oth Male Gen Org	-	-	-	---	-	-	-	---	-	-	-	---
C64	Kidney	8	18.30	0.44	0.21-0.87	1	2.64	0.38	0.05-2.68	9	18.54	0.49	0.25-0.93
C65	Renal Pelvis	1	0.53	1.89	0.26-13.4	1	0.08	12.75	1.79-90.5	2	0.54	3.71	0.92-14.8
C66	Ureter	-	-	-	---	-	-	-	---	-	-	-	---
C67	U Bladder	14	25.14	0.56	0.32-0.94	1	2.03	0.49	0.06-3.50	15	22.72	0.66	0.39-1.09
C68	Unspec Urinary Org	-	-	-	---	-	-	-	---	-	-	-	---
C69	Eye	2	1.64	1.22	0.30-4.88	1	0.54	1.86	0.26-13.1	3	2.18	1.38	0.44-4.27
C70-C72	Brain & Nerv System	8	32.41	0.25	0.12-0.49	3	6.80	0.44	0.14-1.36	11	36.48	0.30	0.16-0.54
C73	Thyroid	10	14.70	0.68	0.36-1.26	6	10.70	0.56	0.25-1.24	16	29.63	0.54	0.33-0.88
C74	Adrenal Glands	2	2.47	0.81	0.20-3.24	-	-	-	---	2	3.27	0.61	0.15-2.44
C75	Oth Endo Gland	-	-	-	---	-	-	-	---	-	-	-	---
C77	Sec Lymph Node	10	14.87	0.67	0.36-1.24	1	2.43	0.41	0.05-2.91	11	15.56	0.71	0.39-1.27
C78	Sec Resp & Dig Org	22	12.33	1.78	1.17-2.70	6	5.50	1.09	0.48-2.42	28	18.89	1.48	1.02-2.14
C79	Oth Sec Org	3	2.99	1.00	0.32-3.10	-	-	-	---	3	3.83	0.78	0.25-2.42
C81	Hodgkins Dis	12	13.77	0.87	0.49-1.53	2	2.42	0.83	0.20-3.30	14	14.69	0.95	0.56-1.60
C82-C85 & C96	NHL	55	47.31	1.16	0.89-1.51	13	8.98	1.45	0.84-2.49	68	51.62	1.32	1.03-1.67
C88	Immunoproli Dis	-	-	-	---	-	-	-	---	-	-	-	---
C90	Mult Myeloma	15	13.16	1.14	0.68-1.89	-	-	-	---	15	14.59	1.03	0.61-1.70
C91	Lymphoid Leuk	29	41.30	0.70	0.48-1.01	6	7.59	0.79	0.35-1.76	35	44.63	0.78	0.56-1.09
C92-C94	Myeloid Leuk	27	42.73	0.63	0.43-0.92	8	10.10	0.79	0.39-1.58	35	50.04	0.70	0.50-0.97
C95	Leukemia Uns	4	5.03	0.79	0.29-2.11	1	1.20	0.83	0.11-5.92	5	5.91	0.85	0.35-2.03
Other & Unspecified Sites		1	1.08	0.92	0.13-6.56	-	-	-	---	1	1.37	0.73	0.10-5.19

**Table 3.** Details of cancer sites and PIR for the cancer sites in the 831 patients with HCV and cancer registered at TMH, Mumbai, India. Year: 2017–2018.

ICD-10	SITE	Male				Female				Total			
		**Observed number**	**Expected number**	**PIR**	**95% CI**	**Observed number**	**Expected number**	**PIR**	**95% CI**	**Observed number**	**Expected number**	**PIR**	**95% CI**
C00	Lip	3	3	1.13	0.36-3.50	1	1	1.36	0.19-9.63	4	3	1.19	0.45-3.17
C01-C02	Tongue	27	38	0.71	0.49-1.03	7	8	0.85	0.41-1.79	34	46	0.74	0.53-1.04
C03-C06	Mouth	51	69	0.74	0.56-0.97	14	13	1.08	0.64-1.83	65	81	0.80	0.63-1.03
C07-C08	Saliv Gland	1	2	0.41	0.06-2.94	-	1	0.00	-	1	4	0.28	0.04-2.02
C09	Tonsil	2	4	0.55	0.14-2.22	-	1	0.00	-	2	4	0.49	0.12-1.97
C10	Oth Oropharynx	5	4	1.38	0.58-3.32	1	0	2.05	0.29-14.56	6	4	1.49	0.67-3.31
C11	Nasopharynx	1	3	0.35	0.05-2.50	2	1	2.29	0.57-9.16	3	4	0.82	0.26-2.53
C12-C13	Hypopharynx	5	9	0.56	0.23-1.34	-	2	0.00	-	5	11	0.47	0.20-1.13
C14	Pharynx	-	0	0.00	#NUM!	-	0	0.00	-	-	0	0.00	-
C15	Oesophagus	17	15	1.12	0.69-1.79	9	8	1.16	0.60-2.23	26	23	1.13	0.77-1.67
C16	Stomach	26	18	1.42	0.96-2.08	8	8	1.04	0.52-2.08	34	26	1.31	0.94-1.84
C17	Small Intestine	3	2	1.65	0.53-5.11	-	1	0.00	-	3	2	1.24	0.40-3.84
C18	Colon	13	12	1.06	0.61-1.82	9	6	1.56	0.81-3.00	22	18	1.23	0.81-1.86
C19-C20	Rectum	14	16	0.87	0.52-1.47	7	7	0.97	0.46-2.03	21	23	0.91	0.59-1.39
C21	Anus	1	2	0.56	0.08-3.98	2	1	2.71	0.68-10.85	3	3	1.20	0.39-3.71
C22	Liver	86	12	7.15	5.79-8.84	30	3	10.42	7.28-14.90	116	15	7.88	6.57-9.45
C23-C24	Gall Bladder	15	18	0.85	0.51-1.40	34	24	1.42	1.02-1.99	49	42	1.17	0.88-1.54
C25	Pancreas	10	8	1.22	0.66-2.28	-	4	0.00	-	10	12	0.83	0.45-1.54
C26	Oth Dig Org	-	0	0.00		-	0	0.00	-	-	0	0.00	-
C30-C31	Nose & Sinus	1	3	0.32	0.04-2.24	-	1	0.00	-	1	4	0.23	0.03-1.65
C32	Larynx	9	13	0.69	0.36-1.34	-	1	0.00	-	9	13	0.67	0.35-1.28
C33-C34	Lung	39	40	0.97	0.71-1.33	15	15	1.01	0.61-1.67	54	55	0.99	0.76-1.29
C37-C38	Oth Thorac Org	1	1	0.83	0.12-5.87	-	0	0.00	-	1	1	0.72	0.10-5.14
C40-C41	Bone	4	9	0.42	0.16-1.13	5	4	1.16	0.48-2.78	9	14	0.66	0.34-1.26
C43	Melanoma of Skin	2	1	1.52	0.38-6.07	-	1	0.00	-	2	2	1.01	0.25-4.04
C44	Other Skin	5	4	1.19	0.49-2.85	1	3	0.40	0.06-2.82	6	7	0.89	0.40-1.99
C45	Mesothelioma	1	1	1.86	0.26-13.18	-	0	0.00	-	1	1	1.50	0.21-10.64
C47&C49	Soft tissue & PNS	7	12	0.57	0.27-1.19	4	7	0.59	0.22-1.58	11	19	0.58	0.32-1.05
C48	Retroperitoneum	2	1	1.65	0.41-6.61	1	1	1.32	0.19-9.34	3	2	1.53	0.49-4.73
C50	Breast	1	1	0.70	0.10-4.98	69	88	0.79	0.62-1.00	70	92	0.76	0.60-0.96
C51	Vulva	-	-	-	-	2	1	2.20	0.55-8.82	2	1	2.14	0.53-8.54
C52	Vagina	-	-	-	-	2	3	0.79	0.20-3.16	2	3	0.77	0.19-3.06
C53	Cervix	-	-	-	-	27	33	0.82	0.56-1.20	27	34	0.80	0.55-1.16
C54-C55	Corpus Uteri	-	-	-	-	13	10	1.37	0.79-2.36	13	10	1.33	0.77-2.28
C56	Ovary etc	-	-	-	-	27	21	1.30	0.89-1.90	27	21	1.26	0.86-1.84
C57	Oth Female Gen Org	-	-	-	-	-	0	0.00	-	-	0	0.00	-
C58	Placenta	-	-	-	-	-	0	0.00	-	-	0	0.00	-
C60	Penis	3	3	0.86	0.28-2.67	-	-	-	-	3	3	0.88	0.28-2.73
C61	Prostate	18	19	0.94	0.59-1.49	-	-	-	-	18	19	0.96	0.61-1.53
C62	Testis	4	6	0.71	0.27-1.90	-	-	-	-	4	5	0.73	0.27-1.94
C63	Oth Male Gen Org	-	0	0.00	-	-	-	-	-	-	0	0.00	-
C64	Kidney	4	9	0.46	0.17-1.22	1	3	0.37	0.05-2.65	5	11	0.44	0.18-1.07
C65	Renal Pelvis	-	0	0.00	-	-	0	0.00	-	-	0	0.00	-
C66	Ureter	-	0	0.00	-	-	0	0.00	-	-	0	0.00	-
C67	U Bladder	14	12	1.17	0.69-1.98	3	2	1.46	0.47-4.53	17	14	1.23	0.76-1.98
C68	Unspec Urinary Org	-	0	0.00	-	-	0	0.00	-	-	0	0.00	-
C69	Eye	-	1	0.00	-	-	1	0.00	-	-	1	0.00	-
C70-C72	Brain & Nerv System	1	15	0.06	0.01-0.46	-	7	0.00	-	1	22	0.05	0.01-0.32
C73	Thyroid	3	7	0.43	0.14-1.33	8	11	0.74	0.37-1.48	11	18	0.61	0.34-1.10
C74	Adrenal Glands	-	1	0.00	-	-	1	0.00	-	-	2	0.00	-
C75	Oth Endo Gland	-	0	0.00	-	1	0	8.82	1.24-62.61	1	0	2.51	0.35-17.84
C77	Sec Lymph Node	8	7	1.13	0.57-2.26	4	2	1.63	0.61-4.33	12	9	1.27	0.72-2.23
C78	Sec Resp & Dig Org	8	6	1.36	0.68-2.73	7	6	1.26	0.60-2.64	15	11	1.31	0.79-2.17
C79	Oth Sec Org	4	1	2.81	1.05-7.48	1	1	1.10	0.16-7.83	5	2	2.15	0.89-5.16
C81	Hodgkins Dis	7	7	1.07	0.51-2.24	2	2	0.82	0.20-3.27	9	9	1.01	0.52-1.94
C82-C85 & C96	NHL	27	23	1.20	0.82-1.75	8	9	0.88	0.44-1.76	35	31	1.12	0.80-1.55
C88	Immunoproli Dis	-	0	0.00	-	-	0	0.00	-	-	0	0.00	-
C90	Mult Myeloma	6	6	0.96	0.43-2.13	-	3	0.00	-	6	9	0.68	0.30-1.51
C91	Lymphoid Leuk	9	20	0.46	0.24-0.88	7	8	0.91	0.43-1.91	16	27	0.59	0.36-0.96
C92-C94	Myeloid Leuk	17	20	0.84	0.52-1.35	9	10	0.88	0.46-1.69	26	30	0.85	0.58-1.26
C95	Leukemia Uns	2	2	0.84	0.21-3.34	-	1	0.00	-	2	4	0.56	0.14-2.23
Other & Unspecified Sites		3	1	5.83	1.88-18.07	-	0	0.00	-	3	1	3.61	1.16-11.19
